# Statins Protect Against Early Stages of Doxorubicin-induced Cardiotoxicity Through the Regulation of Akt Signaling and SERCA2

**DOI:** 10.1016/j.cjco.2022.08.006

**Published:** 2022-08-13

**Authors:** Keith Dadson, Paaladinesh Thavendiranathan, Ludger Hauck, Daniela Grothe, Mohammed Ali Azam, Shanna Stanley-Hasnain, Donya Mahiny-Shahmohammady, Daoyuan Si, Mahmoud Bokhari, Patrick F.H. Lai, Stéphane Massé, Kumaraswamy Nanthakumar, Filio Billia

**Affiliations:** a Toronto General Hospital Research Institute, University of Toronto, Toronto, Ontario, Canada; b Ted Rogers Program in Cardiotoxicity Prevention, Peter Munk Cardiac Centre, University Health Network, University of Toronto, Toronto, Ontario, Canada; c The Hull Family Cardiac Fibrillation Management Laboratory, Toronto General Hospital, Toronto, Ontario, Canada

## Abstract

**Background:**

Doxorubicin-induced cardiomyopathy (DICM) is one of the complications that can limit treatment for a significant number of cancer patients. In animal models, the administration of statins can prevent the development of DICM. Therefore, the use of statins with anthracyclines potentially could enable cancer patients to complete their chemotherapy without added cardiotoxicity. The precise mechanism mediating the cardioprotection is not well understood. The purpose of this study is to determine the molecular mechanism by which rosuvastatin confers cardioprotection in a mouse model of DICM.

**Methods:**

Rosuvastatin was intraperitoneally administered into adult male mice at 100 μg/kg daily for 7 days, followed by a single intraperitoneal doxorubicin injection at 10 mg/kg. Animals continued to receive rosuvastatin daily for an additional 14 days. Cardiac function was assessed by echocardiography. Optical calcium mapping was performed on retrograde Langendorff perfused isolated hearts. Ventricular tissue samples were analyzed by immunofluorescence microscopy, Western blotting, and quantitative polymerase chain reaction.

**Results:**

Exposure to doxorubicin resulted in significantly reduced fractional shortening (27.4% ± 1.11% vs 40% ± 5.8% in controls; *P* < 0.001) and re-expression of the fetal gene program. However, we found no evidence of maladaptive cardiac hypertrophy or adverse ventricular remodeling in mice exposed to this dose of doxorubicin. In contrast, rosuvastatin-doxorubicin-treated mice maintained their cardiac function (39% ± 1.26%; *P* < 0.001). Mechanistically, the effect of rosuvastatin was associated with activation of Akt and phosphorylation of phospholamban with preserved sarcoplasmic/endoplasmic reticulum Ca^2+^ transporting 2 (SERCA2)-mediated Ca^2+^ reuptake. These effects occurred independently of perturbations in ryanodine receptor 2 function.

**Conclusions:**

Rosuvastatin counteracts the cardiotoxic effects of doxorubicin by directly targeting sarcoplasmic calcium cycling.

## Résumé

**Contexte:**

La cardiomyopathie induite par la doxorubicine (CMID) est l’une des complications pouvant limiter le traitement d’un nombre considérable de patients atteints de cancer. Dans des modèles animaux, l’administration de statines peut prévenir l’apparition d’une CMID. Ainsi, l’utilisation de statines avec les anthracyclines pourrait vraisemblablement permettre aux patients de compléter leur chimiothérapie en évitant une cardiotoxicité supplémentaire. Le mécanisme précis qui sous-tend cet effet cardioprotecteur n’est pas entièrement élucidé. Cette étude a pour objectif de déterminer dans un modèle murin de CMID le mécanisme moléculaire par lequel la rosuvastatine confère une cardioprotection.

**Méthodologie:**

La rosuvastatine a été administrée par voie intrapéritonéale à des souris adultes mâles à une dose de 100 μg/kg par jour pendant sept jours, suivie d’une dose unique de doxorubicine de 10 mg/kg administrée par injection intrapéritonéale. Les animaux poursuivaient ensuite le traitement par la rosuvastatine une fois par jour pendant 14 jours supplémentaires. La fonction cardiaque a été mesurée par échocardiographie. Une cartographie optique du calcium a été réalisée sur des cœurs isolés soumis à une perfusion rétrograde selon la méthode de Langendorff. Des échantillons de tissu ventriculaire ont été analysés par microscopie en immunofluorescence, par buvardage de western et par mesure quantitative de l’amplification en chaîne par polymérase.

**Résultats:**

L’exposition à la doxorubicine a entraîné une diminution significative de la fraction de raccourcissement (27,4 % ± 1,11 % vs 40 % ± 5,8 % dans le groupe témoin; *p* < 0,001) et la réexpression du programme génique fœtal. Toutefois, aucune hypertrophie cardiaque inadaptée ni aucun remodelage ventriculaire indésirable n’ont été observés chez les souris ayant été exposées à la dose de doxorubicine étudiée. En revanche, la fonction cardiaque a été préservée chez les souris traitées par l’association rosuvastatine-doxorubicine (39 % ± 1,26 %; *p* < 0,001). Sur le plan du mode d’action, l’effet de la rosuvastatine a été associé à une activation de l’Akt et à une phosphorylation du phospholambane, avec préservation du recaptage de Ca^2+^ médié par la pompe SERCA2 (*sarcoplasmic/endoplasmic reticulum Ca*^*2+*^*transporting 2*). Ces effets sont survenus indépendamment des perturbations de la fonction du récepteur RyR2 (*ryanodine receptor 2*).

**Conclusions:**

La rosuvastatine neutralise les effets cardiotoxiques de la doxorubicine en ciblant directement la circulation sarcoplasmique du calcium.

Doxorubicin (Dox) is a commonly used drug, and its main limiting side effect is dose-dependent risk of cardiomyopathy and heart failure.^1^ The exact mechanism underlying the development of Dox-induced cardiomyopathy (DICM) is not well understood. Previous studies have shown that Dox can generate reactive oxygen species,^2^ interfere with intracellular iron homeostasis,^3^ intercalate into DNA, and inhibit topoisomerase IIβ (Top2β),^4^ all of which contribute to the state of genomic instability that can lead to cardiomyocyte apoptosis.^5^ However, given the failure of antioxidants, β-blockers, and iron chelation therapies in treating DICM, a targetable mechanism underlying DICM has yet to found. Interestingly, abnormalities in the regulation of intracellular calcium (Ca^2+^) cycling are commonly observed in the development of cardiomyopathy, which is also often accompanied by exposure to Dox.^6^, ^7^, ^8^, ^9^

Aside from lowering lipid levels, statins inhibit 3-hydroxy-3-methylglutaryl-coenzyme A reductase^10^, ^11^, ^12^ and exert beneficial effects in the setting of cardiovascular disease. Their pleiotropic effects include the inhibition of the isoprenoid pathway leading to the activation of endothelial nitric oxide synthase through inhibition of the Rho family of small guanosine triphosphatases (GTPases) and attenuation of reactive oxygen species via Rac1 inhibition.^13^^,^^14^ Until recently, the cardioprotective effects of statin pretreatment^2^^,^^15^^,^^16^ have been limited to inhibition of Rac1, 70-kDa heat shock protein (Hsp70) protein levels and receptor for advanced glycation endproducts (RAGE), and activation of high mobility group box 1 (HMBG1) and forkhead box O1 (FOXO1).^17^, ^18^, ^19^, ^20^ Of interest are recent data showing that pretreatment of anoxic H9c2 cells with statins resulted in improved calcium handling through the sarcoplasmic reticulum (SR).^21^

Cardiac performance relies on the balance between Ca^2+^ release and reuptake at the level of the SR. Perturbations in Ca^2+^ homeostasis are important key events in the failing heart.^22^ In the heart, adrenergic signaling leads to activation of protein kinase A (PKA) and Ca^2+^-calmodulin-dependent protein kinase 2 (CaMKII). PKA and CaMKII phosphorylate multiple targets that are important in Ca^2+^ handling, particularly phospholamban (PLN).^23^ In the phosphorylated state, PLN increases the adenosine triphosphate (ATPase) activity of sarcoplasmic/endoplasmic reticulum Ca^2+^ transporting 2 (SERCA2) to enhance Ca^2+^-reuptake into the SR.^24^

Dysregulation of Ca^2+^ handling by Dox has been observed in animal models of DICM.^25^, ^26^, ^27^ Given that Dox can bind directly to SERCA2 and ryanodine receptor 2 (RyR_2_) to disrupt Ca^2+^ transfer between the SR and the cytoplasmic compartments.^28^^,^^29^ we hypothesized that the cardioprotective effect of statins is likely mediated at the level of Ca^2+^ handling and the signaling transduction pathways involved in Ca^2+^ regulation. Herein, we present data that demonstrate that the protective effect of pretreatment with statins is mediated through the maintenance of the Akt/SERCA2 axis for calcium handling, despite exposure to Dox. This effect happens independently of RyR_2_.

## Materials and Methods

### Animal treatment and tissue isolation

All animal usage was in accordance with our institutional animal care guidelines (#4748, Canadian Council in Animal Care). Male C57BL/6J mice (Jackson Labs, Bar Harbor, ME) aged 10-12 weeks (22-26 g) were treated with a single dose of Dox (10 mg/kg intraperitoneally [i.p.] D1515; Sigma-Aldrich, St. Louis, MO). In a subgroup of mice, rosuvastatin (Apotex, Toronto, ON) was given 1 week before Dox at 10 μg/kg per day (10R) or 100 μg/kg per day (100R) i.p. for a further 2 weeks, followed by sacrifice. To study short-term signaling events, mice were starved of food (water given *ad libitum*) for 6 hours and given 1 U/kg insulin (Gibco, Grand Island, NY, 12585-014), i.p. Hearts were isolated 5 or 10 minutes post-insulin administration. Detailed experimental procedures, including echocardiography, confocal immunofluorescence microscopy, terminal deoxynucleotidyl transferase dUTP nick end labeling (TUNEL) assay, Western blotting, densitometry, antibodies, oligonucleotides, and real-time reverse transcriptase quantitative polymerase chain reaction, were performed as described in the Supplemental Appendix S1.

### Optical mapping of calcium transients in a Langendorff setup

Mice were deeply anesthetized with 2%-3% isoflurane, and heparin was injected i.p. Through a mid-transthoracic incision, hearts were extracted. Isolated murine hearts were placed into ice-cold modified Krebs-Henseleit solution containing the following: NaCl, 118 mM; KCl, 4.7 mM; CaCl_2,_ 1.3 mM; MgSO_4,_ 1.2 mM; KH_2_PO_4,_ 1.2 mM; NaHCO_3_, 25 mM;, and glucose, 5.5 mM. The aorta was then cannulated to allow for retrograde perfusion of the coronaries with the modified Krebs-Henseleit solution on a Langendorff apparatus at a temperature of 37°C and pressure at ∼70 mm Hg. After 5-7 minutes of stabilization, the Ca^2+^-sensitive fluorescent dye Rhod2-AM (0.07 μM; Biotium, Fremont, CA) was added slowly over 5 minutes to the circulating perfusion solution for the optical recording of myocardial calcium dynamics. To minimize motion artifacts, a mechanical uncoupler, blebbistatin (1.0 μM; Enzo Life Sciences) was then added slowly over 5 minutes to the perfusion solution. We proceeded with optical mapping at different pacing rates after an additional 3-5 minutes. Dye fluorescence was stimulated using a xenon light source (Moritex Corporation, Saitama, Japan) and a 530-nm green filter (Semrock, IDEX Health & Science, West Henrietta, NY) with the emission light bandpass filtered at 585/40 nm. A high-speed CMOS camera (Ultima-L, Scimedia, Costa Mesa, CA) was used to record dye fluorescence at a sampling rate of 500 frames/second. Image size was 16 mm × 16 mm with a 100 × 100-pixel resolution.

Optical mapping of ventricular epicardial calcium signals was performed using a paced-paused protocol with a Grass S88X stimulator to pace the Langendorff-perfused heart for 30 seconds at 11-13.5 Hz. Fluorescence was recorded at the end of the pacing maneuver. Optical signals recorded during the final 2 seconds of pacing and the first 2 seconds of spontaneous rhythm were analyzed. From an area on the optical maps representing the ventricle, 3 pixels were selected for a feature analysis of Ca^2+^ signals to assess the following calcium transient dynamics.

#### Ca^2+^ amplitude alternans ratio

The Ca^2+^ amplitude alternans ratio was calculated as y/x, where x is the amplitude of higher and y is the amplitude of smaller Ca^2+^ transient amplitude. This measure is unitless, and the absence of alternans is indicated by a ratio of 1. The Ca^2+^ amplitude alternans ratio is an indicator of RyR_2_ function.^30^

#### Spontaneous diastolic Ca^2+^ elevation

Diastolic Ca^2+^ elevation, which is an indicator of RyR_2_ dysfunction^31^ was measured after the 30-second pacing at a different rate before the first spontaneous beat and was normalized to Ca^2+^ amplitude during pacing and expressed as an arbitrary unit (AU).

#### Ca^2+^ transient durations

As an indicator of cytosolic calcium extrusion,^32^ Ca^2+^ transient durations (CaTD) were measured from 0% to 50% of repolarization (CaTD_50_) and from 0% to 80% of repolarization (CaTD_80_), and were expressed in msec, as described previously.^33^ All optical signals acquired in this study were then processed using a custom program written in Matlab (Mathworks, Natick, MA).

### Statistical analysis

All statistical analyses were performed employing GraphPad InStat (version 3.1) and GraphPad Prism (version 8.3.1; GraphPad Software, La Jolla, CA). All data are reported as means ± standard error of the mean (SEM). We considered 2-tailed *P* values of < 0.05 to be significant. When groups passed the normality test, data evaluation between 2 groups was performed by an unpaired Student *t*-test. The statistical significance of 3 groups was calculated using analysis of variance (ANOVA) and the Tukey-Kramer multiple comparison post-test. Additional 2-group ANOVAs were performed between all 2-group combinations when 3-group ANOVA was found to be significant. *P* values were derived from 2-way ANOVA performed for global comparison between groups.

## Results

### Pretreatment with rosuvastatin prevents the development of DICM

**Figure 1:**
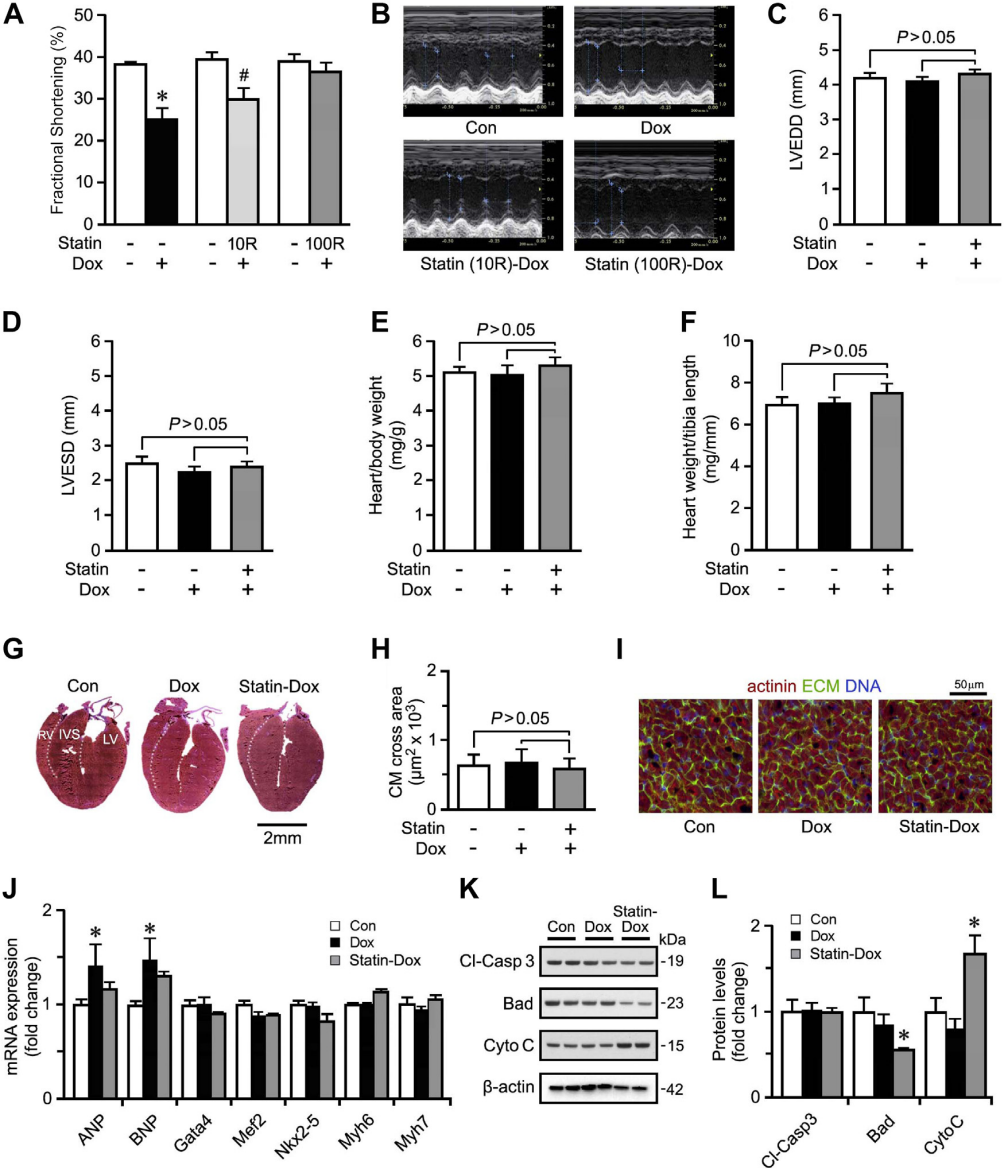
The development of the doxorubicin (Dox)-induced cardiomyopathy model. **(A, B**) Quantification of fractional shortening (**A**) by echocardiography (**B**) in mice treated with Dox or pretreated with rosuvastatin at 10 μg/kg/d (10R) or 100 μg/kg/d (100R) at 14 days post Dox treatment. For the remainder of the study, statin mice were pretreated with 100 μg/kg/d rosuvastatin. n = 6. ∗*P* < 0.01 vs control (Con). (**C, D**) Echocardiographic assessment of cardiac function left ventricular end-diastolic dimension (LVEDD) and left ventricular end-systolic dimension (LVESD), 14 days post-Dox from Control (Con), Dox, and rosuvastatin-treated mice. Data are mean ± standard error of the mean (SEM); n = 6. (**E**) Heart weight corrected for body weight. Data are mean ± SEM. n = 6. (**F**) Heart weight corrected for tibia length. Data are mean ± SEM; n = 6. **(G**) Representative micrographs of Masson trichrome-stained cardiac sections in long-axis view. The **white-yellow dotted line** represents the border between the left ventricle (LV) and the interventricular septum (IVS), and the border between the right ventricle (RV) and the IVS. (**H**, **I**) Quantification (**H**) of cardiomyocyte (CM) cross-sectional area within the LV as analyzed by confocal immunofluorescence microscopy (**I**) at 14 d post-Dox. **Green** = wheat germ agglutinin for extracellular matrix (ECM); **red** = α-actinin; **blue** = 4′,6-diamidino-2-phenylindole for DNA. 100-130 cardiomyocytes quantified from 3 representative images. Data are mean ± SEM; n = 3 mice/group. (**J**) Quantification of mRNA levels from Dox-treated and rosuvastatin-Dox-treatedmice as analyzed by real-time reverse transcriptase quantitative polymerase chain reaction 14 d post-Dox treatment, normalized to β-actin. Data are mean ± SEM; n = 4. ∗*P* < 0.001 vs Con. (**H, I**) Representative immunoblot (**K**) and quantification (**L**) in LV protein extracts from con, Dox-treated, and statin-Dox-treated mice 14 d post-Dox, employing antibodies as indicated on the left (normalized to β-actin). Data are mean ± SEM; n = 3. #*P* < 0.005 Dox vs statin-Dox. ANP, atrial natriuretic peptide; Bad, Bcl-2 associated agonist of cell death; BNP, brain natriuretic peptide; Cl-Casp 3, cleaved caspase-3; CytoC, cytochrome C; Gata 4, GATA binding protein 4; Mef2, myocyte enhancer factor 2; Myh6, myosin heavy chain 6; Nkx2-5, NK2 Homeobox 5. See Supplemental Appendix S2 for original Western Blots.

Several murine models of DICM have been independently reported, with differences in dose and treatment regimens of Dox administered (5-20 mg/kg). When C57BL/6 mice were treated with 20 mg/kg Dox i.p., they developed a significant reduction in fractional shortening (FS) and they succumbed within 1 week (data not shown). In contrast, mice treated with a single injection of 10 mg/kg Dox, a dosage with a relevant LD50,^34^ exhibited 100% survival while developing a significant drop in FS within 3 days (Supplemental Fig. S1; Fig. 1, A-D). FS continued to decline to a nadir by 14 days post-Dox administration (27.4% ± 1.11% vs controls 40% ± 0.58%; *P* < 0.001; Supplemental Fig. S1) that persisted over the long term. Important to note is that pretreatment with rosuvastatin (Statin, 100 μg/kg/day for 7 days) preserved FS, even at 14 days post-Dox (Supplemental Table S1; Fig 1, A-D). Therefore, we employed rosuvastatin at 100 μg/kg per day i.p. for the rest of the study. Notably, a dose of 10 μg/kg per day rosuvastatin did not provide the same benefit.

With the lower FS, no evidence was seen of hypertrophic growth or fibrosis in mice treated with Dox or statin-Dox, in comparison to the control group (Supplemental Table S2; Supplemental Fig. S2; Fig. 1, E-I). However, a partial induction of the fetal gene program occurred, which included atrial natriuretic peptide (ANP) and brain natriuretic peptide (BNP; Fig. 1J) in the hearts of Dox-treated mice. The expression of other cardiac marker genes in mice treated with statin-Dox was comparable to that seen in control animals (Fig. 1J). Thus, the mRNA levels of other stress-response genes remained largely unaltered in the Dox-treated mice. Collectively, these findings indicate that development of DICM does not rely on substantial ventricular remodeling.

Intercalation of Dox into DNA results in double-strand breaks that activate the DNA damage response (DDR). This effect can be evaluated by measuring the phosphorylation status of histone variant γH2AX. We found that Dox treatment led to phosphorylation of Serine at position 139 of γH2AX in cardiomyocytes within 3 days, which resolved by 14 days (Supplemental Fig. S3). This observation was not seen in the statin-Dox-treated mice. Similarly, we detected an early induction of oxidative stress, reflected by the upregulation of mRNA of antioxidant factor catalase at 3 days post-Dox administration that was prevented in the presence of rosuvastatin (Supplemental Fig. S4). Activation of the DDR can contribute to the activation of apoptosis. However, no induction of the pro-apoptotic factors cleaved-caspase 3 or Bcl-2 associated agonist of cell death (Bad) occurred, across all experimental groups. This finding was further supported by the lack of DNA fragmentation as detected by terminal deoxynucleotidyl transferase dUTP nick end labeling (TUNEL) staining (data not shown) and lack of increased protein expression of cleaved caspase 3, BAD or cytochrome C (Fig. 1K and L).

### Rosuvastatin prevents abnormal Ca^2+^ cycling through SERCA2

**Figure 2:**
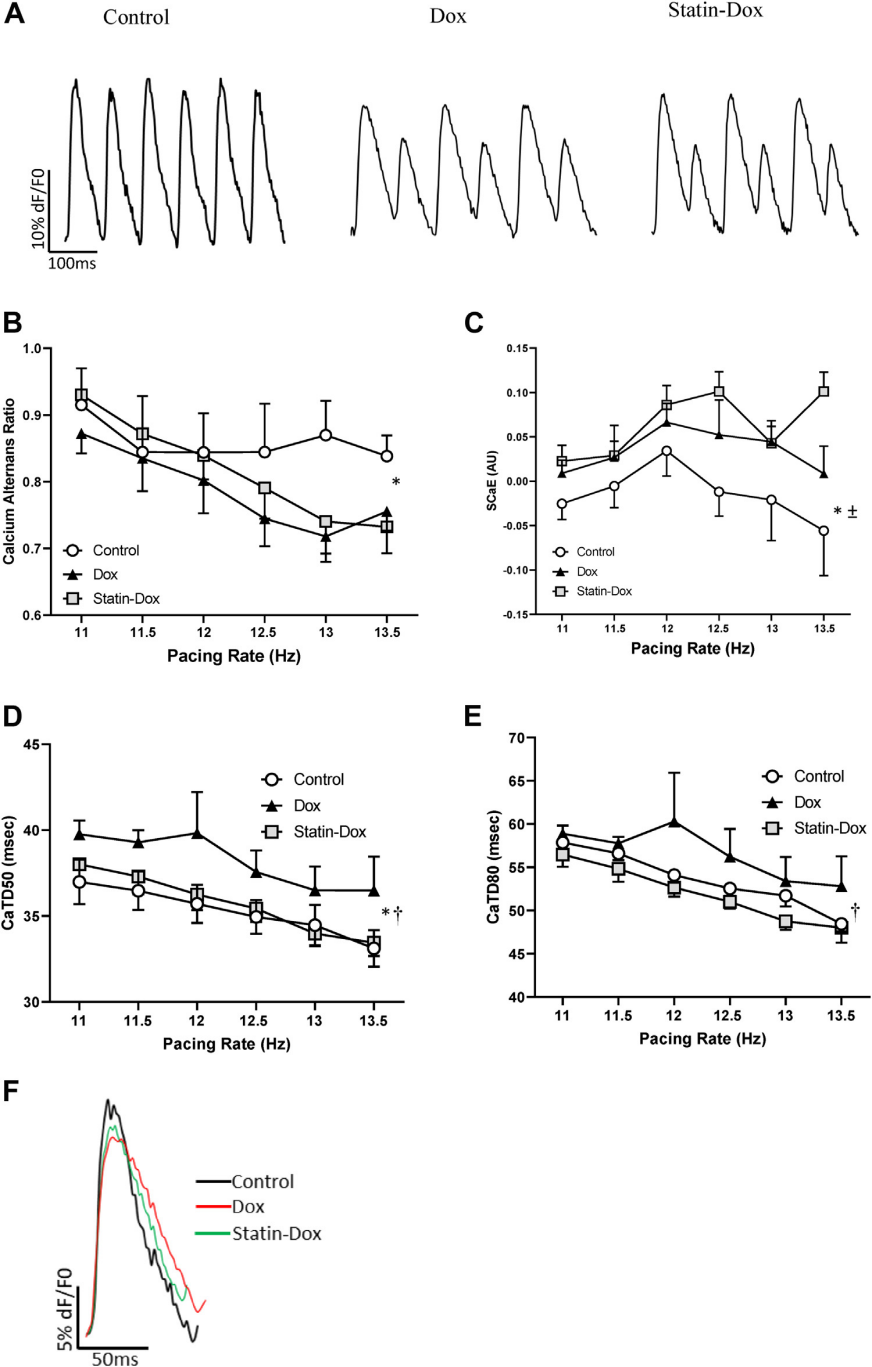
Calcium dysregulation in doxorubicin (Dox)-induced cardiomyopathy that is corrected by rosuvastatin (Statin). (**A**) Representative recordings of ventricular epicardial calcium signals recorded from Langendorff-perfusion hearts at 13 Hz, in Control, Dox, and Statin-Dox groups showing calcium amplitude alternans. (**B**) Calcium amplitude alternans ratio of ventricular epicardial calcium signals at various pacing frequencies. ∗*P =* 0.017 comparing the Con vs Dox groups. (**C**) The spontaneous calcium elevations (SCaEs) in Con, Dox, and Statin-Dox animals at various pacing frequencies. ∗*P =* 0.007, comparing the Con vs Dox groups, and ± *P* < 0.0001, comparing the Con vs Statin-Dox groups. (**D, E**) Repolarization Ca^2+^ transient duration to reach 50% (**D**: calcium transient duration [CaTD]_50_) or 80% (**E**: CaTD_80_) at various pacing frequencies. ∗*P* = 0.004 (CaTD50), comparing the Con vs Dox groups, and ^†^*P* = 0.001 (CaTD50) and ^†^*P* = 0.002 (CaTD80), comparing the Statin-Dox vs Dox groups. (**F**) Representative tracing of superimposed single beat from 3 groups to show the CaTD. All panels were done at various pacing rates and values are mean ± standard error of the mean. *P* values were derived from 2-way analysis of variance performed for global comparison between the groups. For panels **B-F**, we used the following number of animals: Con group n = 5; Dox group n = 10; Statin-Dox group n = 10.

Given that DICM has been associated with disrupted calcium handling (Fig. 2),^26^^,^^28^^,^^35^ we performed optical mapping on Langendorff perfused murine hearts to quantify cardiac Ca^2+^ cycling, *ex vivo*. We observed a significant decrease in the calcium amplitude alternans ratios in Dox-treated hearts compared to controls (Fig. 2A and B; *P =* 00.017). Furthermore, spontaneous calcium elevations were significantly increased in Dox-treated animals (Fig. 2C; *P =* 0.007). Together, these results revealed a Dox-mediated defect in calcium release via the RyR_2_^30^ that is not prevented by rosuvastatin pretreatment.

To determine abnormalities in calcium uptake into the SR via SERCA2a, calcium transient durations at 50% (CaTD_50_) and 80% (CaTD_80_) of maximum were measured. Dox induced a significant prolongation of CaTD50 (*P* = 0.004) and CaTD80 (*P* = 0.129), as compared to control (Fig. 2, D and E). CaTD50 (*P* = 0.001) and CaTD80 (*P* = 0.002) were significantly lower in the statin-Dox-treated group compared to the Dox-treated group (Fig. 2, D and E). These results demonstrate that the impaired Ca^2+^ cycling through SERCA2a is prevented by statin pretreatment.

### Rosuvastatin normalizes doxorubicin-mediated defects in Ca^2+^ signaling

**Figure 3:**
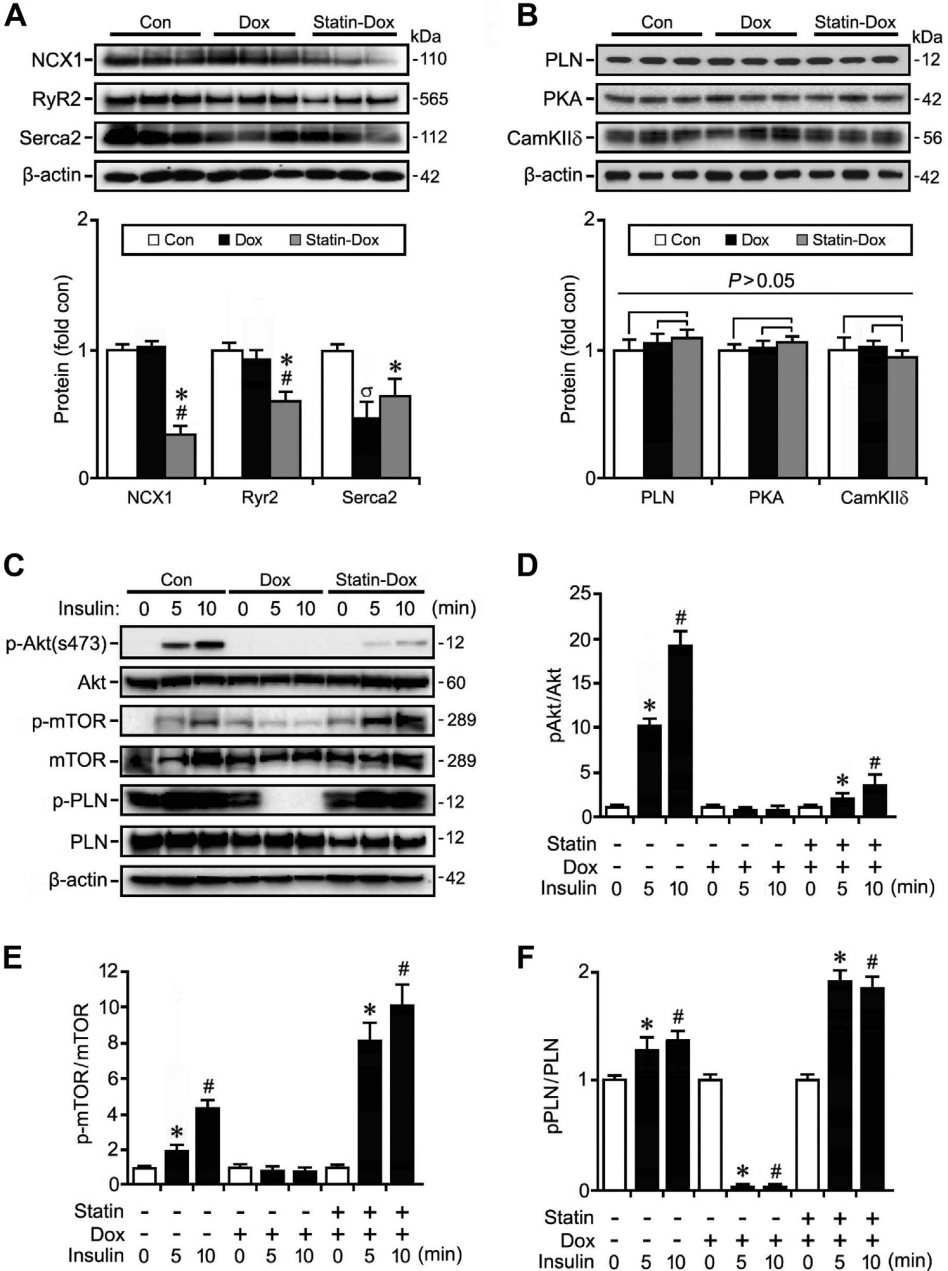
Rosuvastatin (Statin) prevents the inactivation of Akt/ phospholamban (PLN) signaling post-doxorubicin (Dox) treatment. (**A, B**) Immunoblot (**top**) and quantification (**bottom**) of protein expression of intracellular signaling effectors in control (Con), Dox, and Statin-Dox mice at 14 days post-Dox treatment using antibodies as indicated on the left. Western blots were repeated once with similar results. Data are means ± standard error of the mean. n = 3. ∗*P* < 0.01 vs Con ^#^*P* < 0.01 vs Dox. ^σ^*P* < 0.01 vs Con. (**C, D**) Immunoblot (**C**) and quantification (**D-F**) of protein expression of acute changes in intracellular signaling mice after an insulin challenge. Western blots were repeated once done with similar results. Data are means ± standard error of the mean. n = 3. ∗^#^*P* < 0.01 vs 0 min. cam KIId, calcium-calmodulin-dependent protein kinases; mTOR, mammalian target of rapamycin; NCX1, sodium-calcium exchanger 1; p-mTOR, phosphorylated mTOR; PKA, pPLN, phospholamban; RyR2, ryanodine receptor 2; Serca2, sarcoplasmic/endoplasmic reticulum Ca^2+^ transporting 2; S473, serine 473. Supplemental Appendix S2 for original Western Blots.

Next, we examined proteins involved in Ca^2+^ signaling. First, we examined the Na^2+^-Ca^2+^ exchange protein NCX1, which is involved in the extrusion of Ca^2+^ from the cell upon relaxation.^36^^,^^37^ Western blot analysis of left ventricular lysates showed no significant change in protein expression of NCX1 in the Dox-treated group, although NCX1 expression was significantly decreased in the rosuvastatin-Dox-treated group (Fig. 3A), suggesting that changes to intracellular calcium levels could be induced by pretreatment with statins through a possible change in efficiency of calcium handling, rather than protein expression.

Cytosolic calcium concentration is also regulated by the release and uptake of Ca^2+^ from the SR by RyR_2_ and SERCA2, respectively. We found that RyR_2_ expression is similar between the control and Dox-treated groups, but decreased in the rosuvastatin-Dox-treated group (Fig. 3A). In contrast, SERCA2 expression was significantly decreased in the Dox-treated group compared to the control group, and a blunting of this response in SERCA2 expression occurred in the rosuvastatin-Dox groups, although this expression was still statistically decreased as compared to that in the control group (Fig. 3A).

Ca^2+^ reuptake into the SR via SERCA2 is actively regulated by the phosphorylation status of PLN.^24^ We found that total protein expression of PLN was not statistically different between groups (Fig. 3B). However, phosphorylation of PLN was significantly decreased in the Dox-treated group, and pretreatment with statin significantly increased PLN phosphorylation (Fig. 3, C and F). These data suggest that statin pretreatment is associated with enhanced SERCA2 uptake activity. Interestingly, expression of total PKA was decreased in the statin-Dox-treated group, whereas CaMKIIδ expression was decreased by Dox treatment (Fig. 3B). All of these observations suggest that the mechanism of Ca^2+^ handling in our model system was not discernable by measurements of steady-state total protein levels of various components of this pathway.

### Statin restores activation of the Akt/mTor signaling pathway to normalize Ca^2+^ handling

Next, we considered how to trigger the activation of the phosphatidylinositol-3-kinase (Pi3K)/Akt/mammalian target of rapamycin (mTor) signaling involved in regulating Ca^2+^ handling in the heart.^38^ An interesting point to note is that insulin is upstream of this signalling pathway, leading to the activation of Akt.^38^ In keeping with this observation, we were able to demonstrate an enhanced phosphorylation at Ser-473 within 5 minutes of insulin treatment in control mice (Fig. 3, C and D). This enhancement was associated with a concomitant activation of the downstream signalling molecule, mTOR phosphorylation at Ser2448 (Fig. 3, C and E) ^39^

PLN is a downstream target of Akt and may be directly phosphorylated by Akt independently of PKA and CaMKIIδ activation through SR translocation of Akt leading to SR Ca^2+^ uptake.^40^ Akt-dependent Ser16/17-phosphorylation of PLN was detected by Western blot analysis at 5 minutes post-insulin injection (Fig. 3, C and F). In contrast, insulin was unable to induce Akt or mTOR phosphorylation to any appreciable extent in the presence of doxorubicin (Fig. 3, C and D). Phosphorylation of PLN was significantly reduced upon exposure to Dox, but was similar to baseline after 10 minutes of insulin (Fig. 3, C and F). An important finding was that the statin-Dox group had maintenance of Akt/mTor and PLN phosphorylation by insulin at 5 minutes (Fig. 3, C-F). Taking these findings into consideration, we surmise that rosuvastatin pretreatment protects against the development of DICM, at least in part, through the maintenance of the Akt/mTOR/PLN pathway.

## Discussion

Our model of DICM features a sustained decrease in cardiac function with activation of the DDR in cardiomyocytes, and disruptive Akt/PLN signaling leading to impaired calcium handling through RyR_2_ and SERCA2 pathways. Our key finding is that statin pretreatment was able to preserve the Akt/PLN/SERCA2 signaling axis, independent of RyR_2,_ to maintain normal cardiac function. Although DICM has been studied widely, many basic molecular and morphologic questions remain unanswered. In our model, despite activation of the DDR following doxorubicin treatment, cardiomyocyte apoptosis was not observed post-Dox treatment. We concluded that impaired heart function in our model was not driven by cardiomyocyte loss, as reported in other studies, in which even a very low degree of cardiomyocyte apoptosis was sufficient to cause a dilated cardiomyopathy.^41^ Additionally, the lack of early evidence of fibrosis in our model correlates with cardiac magnetic resonance findings in DICM patients, in whom the degree of fibrosis is variable in early disease.^42^^,^^43^ For example, quantitation of fibrosis in response to combined use of rosuvastatin and carvedilol showed an area of fibrosis in the Dox-treated group that was less than 5% of the total area.^44^ Conversely, neither fibrosis nor myofibroblast differentiation was detected in a model of low chronic administration of Dox in mice.^17^ The heterogeneity in these findings may be due to differential treatment schedules and the choice of animal model, whereas cumulative Dox dosage is a strong predictor of adverse outcomes in patients, and cardiac dysfunction and adverse events can occur even at low doses.^45^^,^^46^

Given how well statins are broadly tolerated and the growing need to determine the best therapies in the treatment of DICM, interest in the use of statins in treating DICM has been increasing. Although earlier animal studies using statins showed protection against DICM,^15^^,^^16^^,^^47^ recent studies have given little insight into the underlying mechanism. For example, lovastatin was found to protect against DICM through inhibition of Rac1.^17^ Atorvastatin was shown to inhibit FOXO1 nuclear localization while stabilizing the signal transducer and activator of transcription 3 (STAT3)/specificity protein 1 (Sp1) transcription complex to facilitate promoter binding and transcription of the anti-apoptotic factor survivin and thus reverse the effects of doxorubicin in H9C2 cells.^19^ Atorvastatin was also shown to increase expression of Hsp70^18^, which itself has been shown to protect against DICM.^48^ Interesting to note is that the effects of statins on Akt signaling may be cell-specific, as atorvastatin was shown to upregulate Akt signaling while downregulating caspase-3 in cardiomyocyte cells^18^; conversely, pravastatin and atorvastatin were found to inhibit insulin-dependent Akt phosphorylation in the HepG2, A549, and H1299 cell lines.^49^ Our findings suggest that rosuvastatin preserves Akt-dependent signaling in the heart, where it directly affects calcium homeostasis.

**Figure 4:**
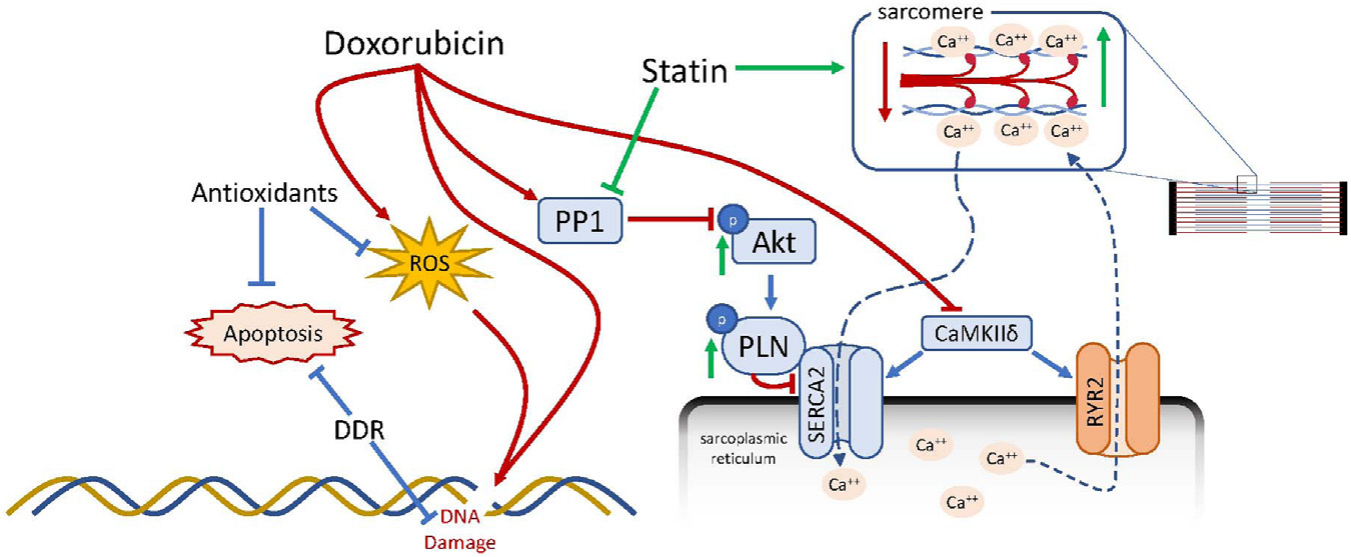
Schematic diagram of mechanisms mediating the protective effect of statins in doxorubicin-induced cardiomyopathy. cam KIId, calcium-calmodulin-dependent protein kinases; DDR, DNA damage response; PLN, phospholamban; PP1, protein phosphatase I; ROS, reactive oxygen species; RyR2, ryanodine receptor 2; Serca2, sarcoplasmic/endoplasmic reticulum Ca^2+^ transporting 2.

The dynamic release and reuptake of calcium from the SR stores are dependent upon RyR_2_ and SERCA2, which are both vital for normal heart function.^50^^,^^51^ Consistent with our study, the involvement of both RyR_2_ and SERCA2 in DICM has been previously shown, although which plays a more principal role remains unclear.^29^^,^^52^ Our detailed analysis confirms that impairment in both RyR_2_ and SERCA2 are present post-Dox treatment. However, statin pretreatment helps to protect against SERCA2 abnormalities to preserve calcium handling, independent of RyR_2_ as illustrated in Figure 4.

Canonical inactivation of PLN suppression on SERCA2 is mediated by step-wise phosphorylation of PLN by PKA at Ser16 followed by phosphorylation at Thr17 by CaMKIIδ.^53^ And, although this mechanism appears to play a principal role in the positive inotropic and lusitropic effects of β-adrenergic agonists,^54^ this effect could be superseded through the translocation of Akt to the SR, where it directly binds and phosphorylates PLN at Thr17 to alleviate SERCA2 suppression.^40^ Certainly, the importance of SERCA2 in DICM has been demonstrated previously, as Dox and its metabolite doxorubicinol have both been shown to interact with and suppress SERCA2 activity and protein expression.^7^^,^^29^ However, transgenic mice overexpressing SERCA2 showed greater sensitivity to Dox-induced damage, suggesting that asymmetric adjustment of Ca^2+^ flux is not beneficial.^55^ In our study, we found that statins preserved left ventricular function through improved SERCA2-mediated Ca^2+^ reuptake, despite the persistence of cardiac alternans and suppressed RyR_2_ protein expression, which could have a long-term impact on heart function. Our study leaves open the possibility that statins could be used in combination with a therapy that specifically targets RyR_2_ to fully restore normal Ca^2+^ handling in the heart. One limitation of our study is that SR calcium load and L-type calcium current, which may play an important role in DICM, were not assessed. In addition, only male mice were assessed. Future work will address the potential for sex-specific differences.

Although other molecular targets have been identified, effective therapies to prevent DICM have yet to be identified. Thus, DICM, once itdevelops, is widely accepted to be irreversible or only partially reversible in the majority of patients.^56^ What makes this even more concerning is that DICM can manifest years following the initial Dox exposure,^56^ which may be too late to effectuate protective therapies. Therefore, interest is ongoing in methods for primary prevention of myocardial injury from anthracycline therapy.

## Limitations

Intact heart calcium transient analysis may provide limited mechanistic insight. Single-cell patch clamping will be ideal for exploring the precise mechanism; this analysis is beyond the scope of this study. However, studies have demonstrated that calcium transient analysis in intact heart could be a surrogate of intracellular calcium dynamics.^30^^,^^32^^,^^57^^,^^58^ Finally, another limitation relates to the use of only males in this study. As data in the literature are conflicting regarding sex as a risk factor for adverse cardiac events in subjects treated with anthracycline,^59^ to simplify the analysis, we examined the early findings of Dox cardiotoxicity in male mice only. Future studies should include both male and female subjects.

## References

[1] Chatterjee K., Zhang J., Honbo N., Karliner J.S. (2010). Doxorubicin cardiomyopathy. Cardiology.

[2] Yoshida M., Shiojima I., Ikeda H., Komuro I. (2009). Chronic doxorubicin cardiotoxicity is mediated by oxidative DNA damage-ATM-p53-apoptosis pathway and attenuated by pitavastatin through the inhibition of Rac1 activity. J Mol Cell Cardiol.

[3] Myers C. (1998). The role of iron in doxorubicin-induced cardiomyopathy. Semin Oncol.

[4] Yang F., Teves S.S., Kemp C.J., Henikoff S. (2014). Doxorubicin, DNA torsion, and chromatin dynamics. Biochim Biophys Acta.

[5] Octavia Y., Tocchetti C.G., Gabrielson K.L. (2012). Doxorubicin-induced cardiomyopathy: from molecular mechanisms to therapeutic strategies. J Mol Cell Cardiol.

[6] Arai M., Tomaru K., Takizawa T. (1998). Sarcoplasmic reticulum genes are selectively down-regulated in cardiomyopathy produced by doxorubicin in rabbits. J Mol Cell Cardiol.

[7] Arai M., Yoguchi A., Takizawa T. (2000). Mechanism of doxorubicin-induced inhibition of sarcoplasmic reticulum Ca(2+)-ATPase gene transcription. Circ Res.

[8] Sag C.M., Kohler A.C., Anderson M.E., Backs J., Maier L.S. (2011). CaMKII-dependent SR Ca leak contributes to doxorubicin-induced impaired Ca handling in isolated cardiac myocytes. J Mol Cell Cardiol.

[9] Azam M.A., Chakraborty P., Bokhari M.M. (2021). Cardioprotective effects of dantrolene in doxorubicin-induced cardiomyopathy in mice. Heart Rhythm Open.

[10] Takemoto M., Node K., Nakagami H. (2001). Statins as antioxidant therapy for preventing cardiac myocyte hypertrophy. J Clin Invest.

[11] Hasegawa H., Yamamoto R., Takano H. (2003). 3-Hydroxy-3-methylglutaryl coenzyme A reductase inhibitors prevent the development of cardiac hypertrophy and heart failure in rats. J Mol Cell Cardiol.

[12] Mital S., Liao J.K. (2004). Statins and the myocardium. Semin Vasc Med.

[13] Adam O., Laufs U. (2014). Rac1-mediated effects of HMG-CoA reductase inhibitors (statins) in cardiovascular disease. Antioxid Redox Signal.

[14] Hauck L., Harms C., Grothe D. (2007). Critical role for FoxO3a-dependent regulation of p21CIP1/WAF1 in response to statin signaling in cardiac myocytes. Circ Res.

[15] Riad A., Bien S., Westermann D. (2009). Pretreatment with statin attenuates the cardiotoxicity of doxorubicin in mice. Cancer Res.

[16] Sharma H., Pathan R.A., Kumar V., Javed S., Bhandari U. (2011). Anti-apoptotic potential of rosuvastatin pretreatment in murine model of cardiomyopathy. Int J Cardiol.

[17] Ohlig J., Henninger C., Zander S. (2018). Rac1-mediated cardiac damage causes diastolic dysfunction in a mouse model of subacute doxorubicin-induced cardiotoxicity. Arch Toxicol.

[18] Gao G., Jiang S., Ge L. (2019). Atorvastatin improves doxorubicin-induced cardiac dysfunction by modulating Hsp70, Akt, and MAPK signaling pathways. J Cardiovasc Pharmacol.

[19] Oh J., Lee B.S., Lim G. (2020). Atorvastatin protects cardiomyocyte from doxorubicin toxicity by modulating survivin expression through FOXO1 inhibition. J Mol Cell Cardiol.

[20] Zhang H., Lu X., Liu Z., Du K. (2018). Rosuvastatin reduces the pro-inflammatory effects of adriamycin on the expression of HMGB1 and RAGE in rats. Int J Mol Med.

[21] Yang Y., Lu X., Rong X. (2015). Inhibition of the mevalonate pathway ameliorates anoxia-induced down-regulation of FKBP12.6 and intracellular calcium handling dysfunction in H9c2 cells. J Mol Cell Cardiol.

[22] Yano M., Ikeda Y., Matsuzaki M. (2005). Altered intracellular Ca2+ handling in heart failure. J Clin Invest.

[23] Gray C.B., Heller Brown J. (2014). CaMKIIdelta subtypes: localization and function. Front Pharmacol.

[24] Park W.J., Oh J.G. (2013). SERCA2a: a prime target for modulation of cardiac contractility during heart failure. BMB Rep.

[25] Jensen R.A. (1986). Doxorubicin cardiotoxicity: contractile changes after long-term treatment in the rat. J Pharmacol Exp Ther.

[26] Ondrias K., Borgatta L., Kim D.H., Ehrlich B.E. (1990). Biphasic effects of doxorubicin on the calcium release channel from sarcoplasmic reticulum of cardiac muscle. Circ Res.

[27] Pessah I.N., Durie E.L., Schiedt M.J., Zimanyi I. (1990). Anthraquinone-sensitized Ca2+ release channel from rat cardiac sarcoplasmic reticulum: possible receptor-mediated mechanism of doxorubicin cardiomyopathy. Mol Pharmacol.

[28] Saeki K., Obi I., Ogiku N. (2002). Doxorubicin directly binds to the cardiac-type ryanodine receptor. Life Sci.

[29] Hanna A.D., Lam A., Tham S., Dulhunty A.F., Beard N.A. (2014). Adverse effects of doxorubicin and its metabolic product on cardiac RyR2 and SERCA2A. Mol Pharmacol.

[30] Sun B., Wei J., Zhong X. (2018). The cardiac ryanodine receptor, but not sarcoplasmic reticulum Ca(2+)-ATPase, is a major determinant of Ca(2+) alternans in intact mouse hearts. J Biol Chem.

[31] Dridi H., Kushnir A., Zalk R. (2020). Intracellular calcium leak in heart failure and atrial fibrillation: a unifying mechanism and therapeutic target. Nat Rev Cardiol.

[32] Jaimes R., Walton R.D., Pasdois P. (2016). A technical review of optical mapping of intracellular calcium within myocardial tissue. Am J Physiol Heart Circ Physiol.

[33] Azam M.A., Zamiri N., Masse S. (2017). Effects of late sodium current blockade on ventricular refibrillation in a rabbit model. Circ Arrhythm Electrophysiol.

[34] Aston W.J., Hope D.E., Nowak A.K. (2017). A systematic investigation of the maximum tolerated dose of cytotoxic chemotherapy with and without supportive care in mice. BMC Cancer.

[35] Zhang Y., Chen Y., Zhang M. (2014). Doxorubicin induces sarcoplasmic reticulum calcium regulation dysfunction via the decrease of SERCA2 and phospholamban expressions in rats. Cell Biochem Biophys.

[36] Kim J.J., Yang L., Lin B. (2015). Mechanism of automaticity in cardiomyocytes derived from human induced pluripotent stem cells. J Mol Cell Cardiol.

[37] Acsai K., Antoons G., Livshitz L., Rudy Y., Sipido K.R. (2011). Microdomain [Ca(2)(+)] near ryanodine receptors as reported by L-type Ca(2)(+) and Na+/Ca(2)(+) exchange currents. J Physiol.

[38] Shiojima I., Walsh K. (2006). Regulation of cardiac growth and coronary angiogenesis by the Akt/PKB signaling pathway. Genes Dev.

[39] Sciarretta S., Volpe M., Sadoshima J. (2014). Mammalian target of rapamycin signaling in cardiac physiology and disease. Circ Res.

[40] Catalucci D., Latronico M.V., Ceci M. (2009). Akt increases sarcoplasmic reticulum Ca2+ cycling by direct phosphorylation of phospholamban at Thr17. J Biol Chem.

[41] Wencker D., Chandra M., Nguyen K. (2003). A mechanistic role for cardiac myocyte apoptosis in heart failure. J Clin Invest.

[42] Raj S., Franco V.I., Lipshultz S.E. (2014). Anthracycline-induced cardiotoxicity: a review of pathophysiology, diagnosis, and treatment. Curr Treat Options Cardiovasc Med.

[43] Carvalho F.S., Burgeiro A., Garcia R. (2014). Doxorubicin-induced cardiotoxicity: from bioenergetic failure and cell death to cardiomyopathy. Med Res Rev.

[44] Kim Y.H., Park S.M., Kim M. (2012). Cardioprotective effects of rosuvastatin and carvedilol on delayed cardiotoxicity of doxorubicin in rats. Toxicol Mech Methods.

[45] Swain S.M., Whaley F.S., Ewer M.S. (2003). Congestive heart failure in patients treated with doxorubicin: a retrospective analysis of three trials. Cancer.

[46] Cardinale D., Colombo A., Bacchiani G. (2015). Early detection of anthracycline cardiotoxicity and improvement with heart failure therapy. Circulation.

[47] Ramanjaneyulu S.V., Trivedi P.P., Kushwaha S., Vikram A., Jena G.B. (2013). Protective role of atorvastatin against doxorubicin-induced cardiotoxicity and testicular toxicity in mice. J Physiol Biochem.

[48] Zerikiotis S., Angelidis C., Dhima I. (2019). The increased expression of the inducible Hsp70 (HSP70A1A) in serum of patients with heart failure and its protective effect against the cardiotoxic agent doxorubicin. Mol Cell Biochem.

[49] Roudier E., Mistafa O., Stenius U. (2006). Statins induce mammalian target of rapamycin (mTOR)-mediated inhibition of Akt signaling and sensitize p53-deficient cells to cytostatic drugs. Mol Cancer Ther.

[50] Swift F., Franzini-Armstrong C., Oyehaug L. (2012). Extreme sarcoplasmic reticulum volume loss and compensatory T-tubule remodeling after Serca2 knockout. Proc Natl Acad Sci U S A.

[51] Bround M.J., Asghari P., Wambolt R.B. (2012). Cardiac ryanodine receptors control heart rate and rhythmicity in adult mice. Cardiovasc Res.

[52] Todorova V.K., Siegel E.R., Kaufmann Y. (2020). Dantrolene attenuates cardiotoxicity of doxorubicin without reducing its antitumor efficacy in a breast cancer model. Transl Oncol.

[53] Luo W., Chu G., Sato Y. (1998). Transgenic approaches to define the functional role of dual site phospholamban phosphorylation. J Biol Chem.

[54] MacLennan D.H., Kranias E.G. (2003). Phospholamban: a crucial regulator of cardiac contractility. Nat Rev Mol Cell Biol.

[55] Burke B.E., Olson R.D., Cusack B.J., Gambliel H.A., Dillmann W.H. (2003). Anthracycline cardiotoxicity in transgenic mice overexpressing SR Ca2+-ATPase. Biochem Biophys Res Commun.

[56] Thavendiranathan P., Nolan M.T. (2017). An emerging epidemic: cancer and heart failure. Clin Sci (Lond).

[57] Lang D., Holzem K., Kang C. (2015). Arrhythmogenic remodeling of beta2 versus beta1 adrenergic signaling in the human failing heart. Circ Arrhythm Electrophysiol.

[58] Chen P.S., Ogawa M., Maruyama M. (2012). Imaging arrhythmogenic calcium signaling in intact hearts. Pediatr Cardiol.

[59] Meiners B., Shenoy C., Zordoky B.N. (2018). Clinical and preclinical evidence of sex-related differences in anthracycline-induced cardiotoxicity. Biol Sex Differ.

