# Supplementary material for: Statins Protect Against Early Stages of Doxorubicin-induced Cardiotoxicity Through the Regulation of Akt Signaling and SERCA2
Source: CJC Open. 2022 Aug 13;4(12):1043–52. doi: 10.1016/j.cjco.2022.08.006 (PMC9764135; doi:10.1016/j.cjco.2022.08.006)

SUPPLEMENTARY MATERIAL

Supplemental Appendix S1. Supplemental Materials and Methods

Echocardiography

Echocardiography on anesthetized mice (2.0% isoflurane, 98% oxygen) was performed using a 15-MHz linear ultrasound transducer (Vivid7; GE). Body temperature was maintained at 37°C. M-mode measurements of the LV end-diastolic diameter (LVEDD) and LV end-systolic diameter (LVESD) were made in triplicate from short-axis views at the level of the papillary muscle and averaged over three to six beats. LVEDD was measured at the time of the apparent maximal LV diastolic dimension, whereas LVESD was measured at the time of the most anterior systolic excursion of the posterior wall. LV fractional shortening (FS) was calculated as follows: FS = (LVEDD − LVESD)/LVEDD × 100%.

Immunofluorescence microscopy and apoptosis assay

Mice were euthanized without anesthesia by cervical dislocation between 9-11am. Anticoagulant was not administered. Hearts were quickly excised and rinsed in 20 ml ice-cold PBS, pH 7.4 without Ca^{2+}/Mg^{2+} (no. 10010023; Thermo Fisher Scientific), fixed in 4% PBS-buffered formalin for 50 min at room temperature with constant agitation and incubated in 0.3 M glycine in PBS (pH 7.4; 10 ml) at 4°C for 3 to 5 days. After embedding hearts in Tissue-Tek OCT Compound (Sakura, Finetek; VWR) sections were cut at 10 μm thickness using a cryostat (HM525 NX; Thermo Fisher Scientific) and mounted on histological slides (no. 48311-703; VWR). Detection of fragmented genomic DNA was performed by terminal deoxynucleotidyl transferase-mediated dUTP nick-end-labeling (TUNEL) according to the manufacturer’s instructions (1684795910; Roche). These samples were co-stained with cardiac-specific anti-sarcomeric α-actinin after permeabilization. Specimen were permeabilized in 1.0% Triton X-100 in TBS composed of 20 mM Tris, 150 mM NaCl, pH 7.6 for 60 minutes at room temperature. Samples were incubated with primary antibodies (listed in Table 1 in TBS/1.0% Triton X-100, for 16-20 hours at room temperature without

agitation. Thereafter, the specimen were briefly rinsed with TBS. For visualization, samples were incubated with secondary antibodies, diluted 150-fold in TBS/1.0% Triton X-100 for 45 min in the dark (Alexa Fluor 488-goat anti-rat IgG, A-11006; Alexa Fluor 555-goat anti-rabbit IgG, A-21429; Alexa Fluor 555-goat anti-mouse IgG, A-2142; Alexa Fluor 647-goat anti-mouse IgG, A-20990 (Thermo Fisher Scientific). Specimen were briefly rinsed with TBS, and nuclear DNA was visualized with 4,6-diamidino-2-phenylindole (Dapi; 1.0 µg/ml in PBS) (D9542; Sigma-Aldrich). ProLong Diamond antifade reagent (no. 36965; Thermo Fisher Scientific) was applied, and samples were sealed with a coverslip. Three-dimensional confocal laser scanning microscopy was performed on a Zeiss LSM700 confocal microscope and LSM Zen 2009 data acquisition software (AOMF-Advanced Optical Microscopy Facility, Ontario Cancer Institute, Toronto, ON Canada). For determination of cardiomyocyte cell size and ventricular remodeling, ventricular samples were stained with sarcomeric α-actinin and Alexa Fluor 488-conjugated wheat germ agglutinin (WGA) (W11261; Thermo Fisher Scientific). Cross-dimensions of adult cardiomyocytes and fibrotic area were determined by planimetry of immunofluorescence microphotographs using ImageJ (Version 1.51d; National Institutes of Health, Bethesda, MD, https://imagej.nih.gov/ij/). After recording, simple adjustments and assembly of entire and cropped microphotographs were performed employing Adobe Photoshop CS6.

Preparation of protein extracts from left ventricular heart tissue extracts

Individual left ventricular (LV) specimens were rapidly cut into 4-6 pieces, briefly rinsed in 40 ml ice-cold PBS, immediately snap frozen and stored at -80°C. S To prepare total protein extracts, LV tissue (80-100 mg wet weight) was placed in a petri dish, kept on ice and was minced into 1-2 mm cubes for 60 sec using a scalpel. The minced tissue was transferred to a 1.5 ml reaction tube and resuspended in 1.5 ml ice-cold RIPA Buffer (no. 9806; Cell Signaling) that was further supplemented with 1,4-Dithiothreitol (4 mM final concentration; no. 10197777001; Sigma) and

protease/phosphatase inhibitors (HALT^{TM} Protease and Phosphatase Inhibitor Cocktail; no. 78440; Thermo Fisher Scientific). Cells were lysed at 4ºC for 3 minutes at a frequency of 30/sec employing the TissueLyser II (Qiagen). Samples were incubated on ice for 30 minutes and vortexed every 5 minutes for 30 seconds. Samples were then subjected to 10 cycles of 3 seconds on/1 second off sonification at 30% amplitude on ice, and incubated for 10 minutes, and vortexed every 5 minutes for 30 seconds. After centrifugation at 12,700 rpm for 30 minutes at 4ºC, the supernatant was mixed with 10% glycerol, aliquoted, snap frozen on dry ice, and stored at -80°C. Total protein concentration was determined employing the Qubit™ Protein Assay Kit (no. 33211; Thermo Fisher Scientific) and a Qubit™ 2.0 Fluorometer (Thermo Fisher Scientific).

Western blotting

Protein samples were mixed with equal amounts of sample buffer (Novex Bolt LDS sample buffer, no. 2107345; Thermo Fisher Scientific) and boiled for 7 minutes. Samples (60 mg total protein per lane) were resolved (Novex Bolt MES SDS-Running Buffer, 2122845; Thermo Fisher Scientific) by SDS-PAGE employing 4-12% pre-cast gradient gels (Novex Bolt 4-12% Bis-Tris Plus; no. NW04120BOX; Thermo Fisher Scientific). To monitor protein migration, protein transfer onto membranes and sizing of protein, prestained protein markers (Seeblue Plus2; LC5925; Thermo Fisher Scientific) were run simultaneously with the samples during SDS-polyacrylamide gel electrophoresis.

Samples were electrotransferred for 7 minutes employing semi-dry iBlot PVDF Gel transfer Stacks (no. 2020-07-16; Thermo Fisher Scientific) and an iBlot system (Thermo Fisher Scientific). Detection was performed with Luminata Crescendo (WBLUR0100, Millipore) on a digital imaging system (Molecular Imager ChemiDoc Imaging System; BioRad). We employed the following secondary antibodies for chemiluminescence detection of proteins: horseradish

peroxidase (HRP)-conjugated anti-rabbit IgG (no. 7074; Cell Signaling), HRP-conjugated anti-mouse IgG (no. 7076; Cell Signaling).

Antibodies employed for IF and WB:

Gene name	Catalog No.	Vendor	Application	Dilution Factor
**β-actin**	A7607	Sigma	WB	1000
**α-actinin, sarcomeric**	A7811	Sigma	IF	50
**Akt**	4691	Cell Signaling	WB	1000
**p-Akt(s473)**	4060	Cell Signaling	WB	1000
**Bad**	9239	Cell Signaling	WB	1000
**CamkIIγ**	PA5-22168	Fisher	WB	1000
**Cl-Casp3**	9664S	Cell Signaling	WB	1000
**CytoC**	sc-13560	SantaCruz	WB	1000
**mTor**	2983	Cell Signaling	WB	1000
**p-mTor**	5536	Cell Signaling	WB	1000
**NCX1**	79350	Cell Signaling	WB	500
**PKA**	5842	Cell Signaling	WB	1000
**PLN**	14562	Cell Signaling	WB	1000
**p-PLN**	8496	Cell Signaling	WB	1000
**Ryr2**	PA5-38329	Fisher	WB	1000
**Serca2**	9580	Cell Signaling	WB	500

IF, immunofluorescence microscopy. WB, Western blot.

Total RNA isolation, reverse transcription, and quantitative real time PCR assays

We carried out two-step reverse transcriptase (RT) and quantitative real-time polymerase chain reactions (qPCR) on a LightCycler 480 (Roche; TMDT Core Facility) for mRNA analysis. Total RNA from mouse and human ventricular cardiac specimen was isolated with Trizol reagent (no. 15596026; Thermo Fisher Scientific), and Phase lock Gels (no. 826754; VWR) were employed to

eliminate interphase-protein contaminations. We used 500 ng total RNA in a 20 µl reaction for first-strand cDNA synthesis employing the SensiFast cDNA synthesis kit (BIO-65053; Bioline). For qPCR, we employed 4.0 µl first-strand synthesis product, diluted 5-fold with MilliQ-grade water and the Quanta Accustart II PCR Supermix (no, 95136-04; VWR) with EvaGreen dye (no. 31000; VWR). Relative quantification of transcript levels was performed using the ΔΔCt method with normalization to β-actin employing the data analysis module in conjunction with the ΔΔCt method.

Primer sequences used in RT-qPCR assays:

Gene Symbol	Forward	Reverse
**ANP**	GCTTCCAGGCCATATTGGAG	GGGGGCATGACCTCATCTT
**BNP**	GAGGTCACTCCTATCCTCTGG	GCCATTTCCTCCGACTTTTCTC
**Gata4**	CCCTACCCAGCCTACATGG	ACATATCGAGATTGGGGTGTCT
**Mef2a**	CAGGTGGTGGCAGTCTTGG	TGCTTATCCTTTGGGCATTCAA
**Nkx2-5**	GACAAAGCCGAGACGGATGG	CTGTCGCTTGCACTTGTAGC
**α-MHC**	GCCCAGTACCTCCGAAAGTC	GCCTTAACATACTCCTCCTTGTC
**β-MHC**	ACTGTCAACACTAAGAGGGTCA	TTGGATGATTTGATCTTCCAGGG

Supplemental Figure S1.

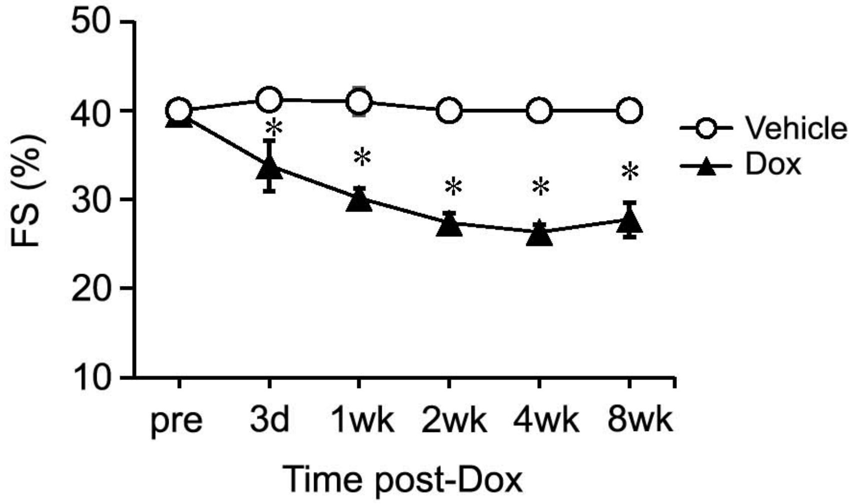

Supplementary Figure S1: Dox-treatment results in a change in fractional shortening (FS) in mice as analzyed by echocardiographic measurements. Mice were 12-14 weeks of age at the initiation of treatment. Data are means±s.e.m. *n*=6. **P*<0.05 vs. vehicle.

Supplemental Figure S2.

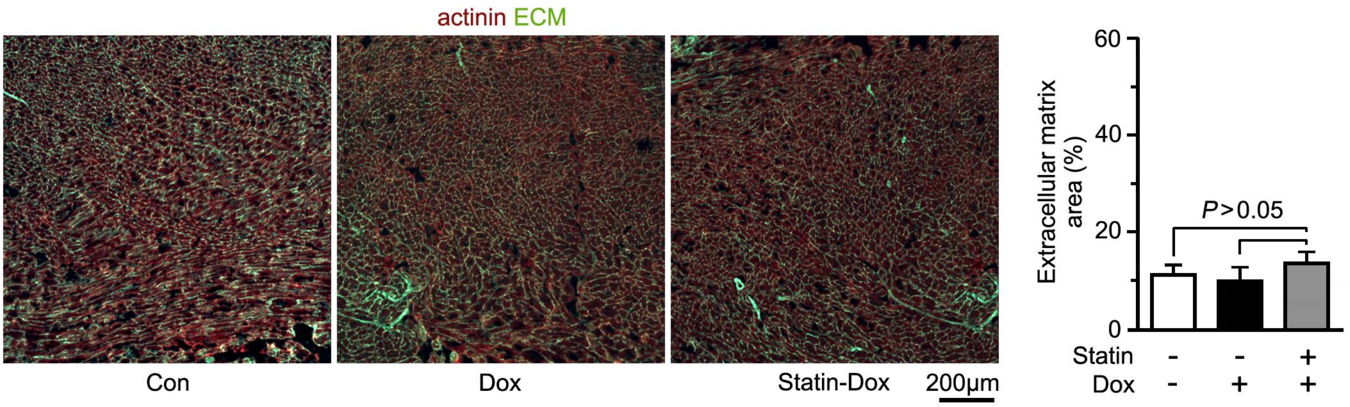

Supplementary Figure S2: Analysis of fibrosis in longitudinal myocardial left ventricular tissue sections by immunofluorescence microscopy (left panel) employing WGA staining (green) of the extracellular matrix and cytoplasmic cardiomyocyte-specific anti-actinin (red). Quantification of fibrosis in cardiac specimen (right panel). Data are mean±s.e.m. n=4. n.s., not significant.

Supplemental Figure S3.

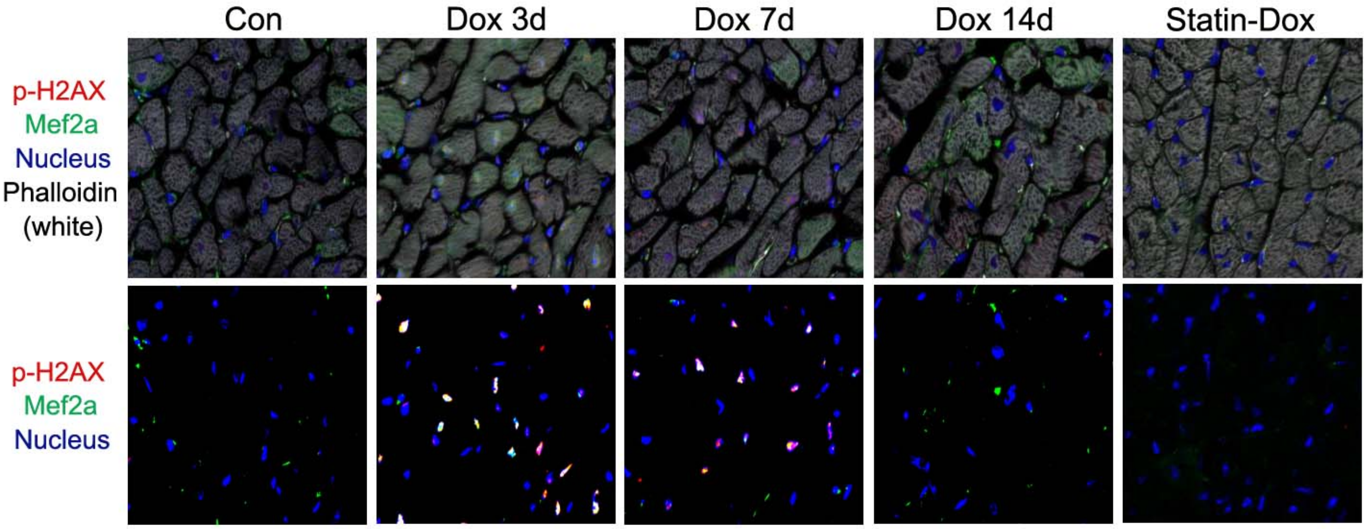

Supplementary Figure S3: Representative confocal immunofluorescence microscopy at 0d-14d post-Dox. Red, phospho-H2Ax. Green, MEF2a for cardiomyocyte-specific transcription factor. Blue, Dapi for DNA. White, actin. Dox, doxorubicin. Statin-Dox, 100 mg/kg/d rosuvastatin + 10 mg/kg doxorubicin.

Supplemental Figure S4.

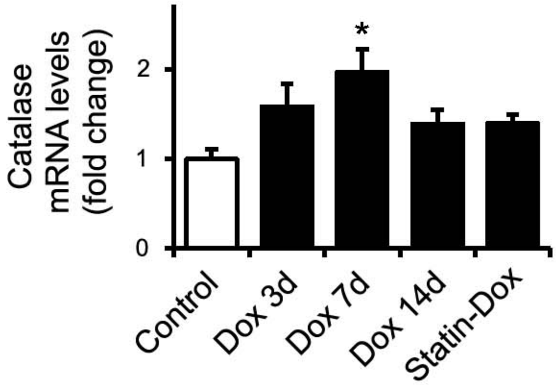

Supplementary Figure S4: Quantification of mRNA levels for the anti-oxidant factor catalase that is differentially regulated post-Dox. Data are means±s.e.m. *n*=4. **P*<0.01 vs. control.

Supplemental Table S1: Echocardiographic measurements for wild-type C57BL/6 mice.
Group 1: Controls

LVEDD (mm)	LVESD (mm)	IVSd (mm)	LVPWd (mm)
4.23	2.38	0.57	0.65
4.14	2.31	0.67	0.58
4.24	2.61	0.65	0.65
4.31	2.43	0.61	0.61
4.14	2.58	0.58	0.56
4.18	2.29	0.63	0.58

LVEDD, left ventricular end-diastolic dimension. LVESD, left ventricular end-systolic dimension. PWD, posterior wall diastolic. IVSd, Interventricular septum thickness at end-diastole. LWPWD, Left ventricular posterior wall thickness at end-diastole.

Group 2: Doxorubicin

LVEDS (mm)	LVESD (mm)	IVSd (mm)	LVPWd (mm)
4.11	2.18	0.68	0.61
4.21	2.35	0.54	0.65
4.49	2.21	0.66	0.59
4.29	2.46	0.69	0.63
4.01	2.28	0.58	0.65
3.98	2.06	0.62	0.58

Group 3: Dox/Statin

LVEDD (mm)	LVESD (mm)	IVSd (mm)	LVPWd (mm)
4.38	2.66	0.83	0.83
3.92	2.72	0.74	0.81
4.47	2.31	0.85	0.76
4.51	2.26	0.79	0.85
3.77	2.18	0.76	0.80
3.92	2.39	0.79	0.83

Supplemental Table S2: HBW and HTL for wild-type C57BL/6 mice.

Group 1: Controls

Heart weight [mg]	Body weight [g]	HBW	Tibia length [mm]	HTL
122	24.0	5.1	18.9	6.5
147	26.6	5.5	19.3	7.6
128	25.8	5.0	18.3	7.0
109	20.1	5.5	15.8	6.9
119	23.3	5.1	16.1	7.4
113	21.6	5.3	15.5	7.3

HBW, heart/body weight ratio. HTL, heart/tibia length ratio.

Group 2: Doxorubicin

Heart weight [mg]	Body weight [g]	HBW	Tibia length [mm]	HTL
119	22.5	5.3	21.3	7.3
126	22.9	5.5	19.1	6.9
117	22.5	5.2	18.9	7.2
142	28.9	4.9	18.8	7.3
132	26.4	5.0	18.9	7.4
129	27.5	4.7	19.2	6.8

Group 3: Statin/Dox

Heart weight [mg]	Body weight [g]	HBW	Tibia length [mm]	HTL
127	22.7	5.6	17.2	7.4
129	24.8	5.2	17.9	7.2
105	21.9	4.8	13.4	7.8
140	25.4	5.5	19.4	7.2
140	24.1	5.8	19.7	7.1
133	26.0	5.1	17.3	7.7

Supplemental Appendix S2. Original Western Blots

Uncropped WB shown in Figure 1L:

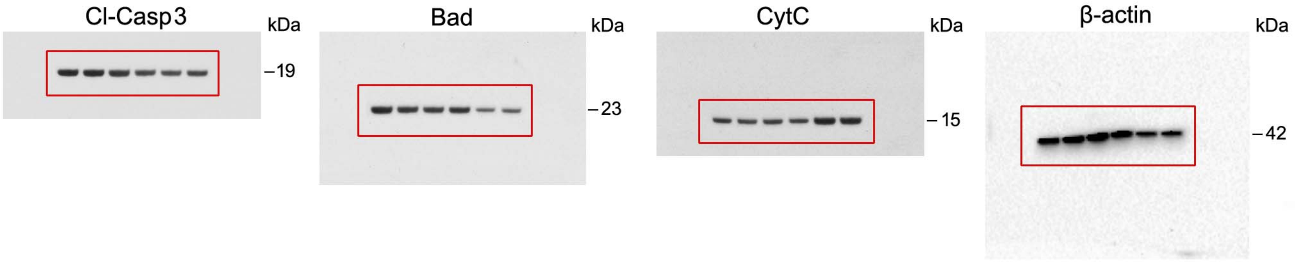

Uncropped WB shown in Figure 3A

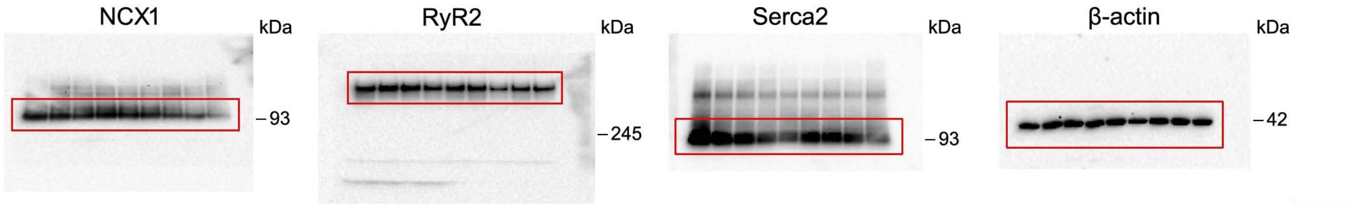

Uncropped WB shown in Figure 3B

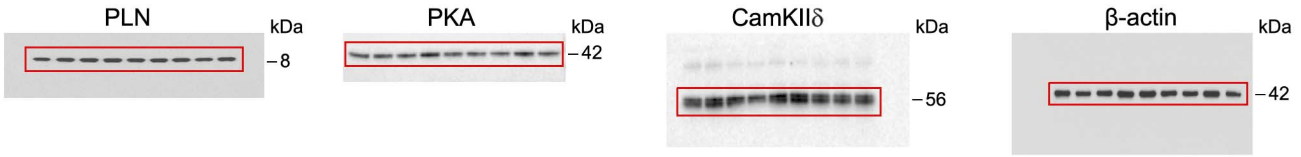

Uncropped WB shown in Figure 3C

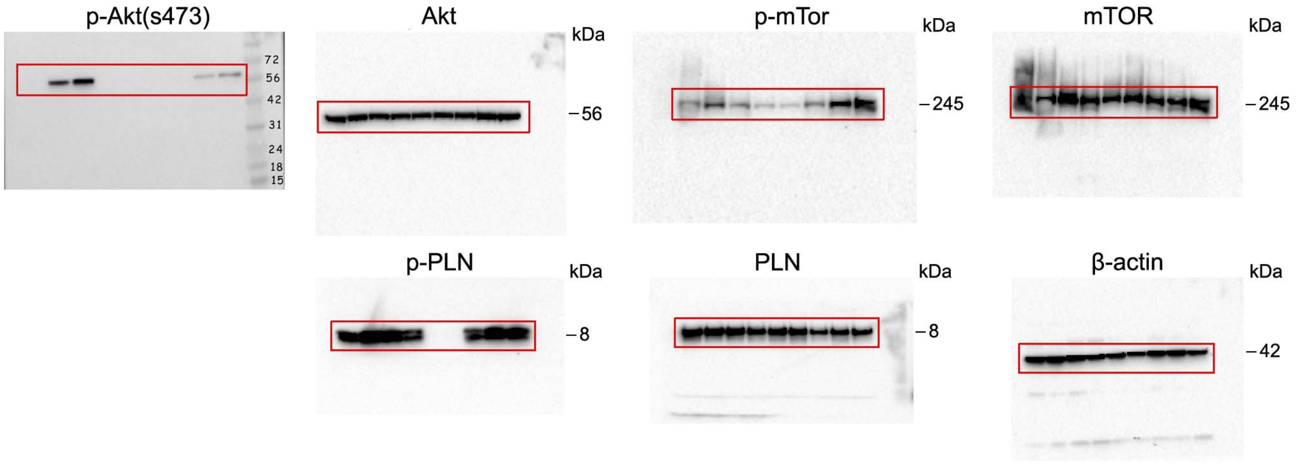

Supplement: Supplementary Material [file mmc1.pdf]
